# Actin Modulation Regulates the Alpha-1-Syntrophin/p66Shc Mediated Redox Signaling Contributing to the RhoA GTPase Protein Activation in Breast Cancer Cells

**DOI:** 10.3389/fonc.2022.841303

**Published:** 2022-02-21

**Authors:** Roshia Ali, Hilal Ahmad Mir, Rabia Hamid, Basharat Bhat, Riaz A. Shah, Firdous A. Khanday, Sahar Saleem Bhat

**Affiliations:** ^1^ Department of Biotechnology, University of Kashmir, Srinagar, India; ^2^ Department of Biochemistry, University of Kashmir, Srinagar, India; ^3^ Department of Nanotechnology, University of Kashmir, Srinagar, India; ^4^ National Agricultural Higher Education Project (NAHEP) Sher-e-Kashmir University of Agricultural Sciences and Technology-Kashmir, Srinagar, India; ^5^ Division of Animal Biotechnology, Sher-e-Kashmir University of Agricultural Sciences and Technology-Kashmir, Faculty of Veterinary Sciences and Animal Husbandry, Srinagar, India

**Keywords:** Actin, Alpha-1-Syntrophin, p66Shc, ROS, RhoA, Breast Cancer, Cytochalasin D

## Abstract

SNTA1 signaling axis plays an essential role in cytoskeletal organization and is also implicated in breast cancers. In this study, we aimed to investigate the involvement of actin cytoskeleton in the propagation of SNTA1/p66shc mediated pro-metastatic cascade in breast cancer cells.The effect of actin filament depolymerization on SNTA1-p66Shc interaction and the trimeric complex formation was analyzed using co-immunoprecipitation assays. Immunofluorescence and RhoA activation assays were used to show the involvement of SNTA1-p66Shc interaction in RhoA activation and F-actin organization. Cellular proliferation and ROS levels were assessed using MTT assay and Amplex red catalase assay. The migratory potential was evaluated using transwell migration assay and wound healing assay.We found that cytochalasin D mediated actin depolymerization significantly declines endogenous interaction between SNTA1 and p66Shc protein in MDA-MB-231 cells. Results indicate that SNTA1 and p66Shc interact with RhoA protein under physiological conditions. The ROS generation and RhoA activation were substantially enhanced in cells overexpressing SNTA1 and p66Shc, promoting proliferation and migration in these cells. In addition, we found that loss of SNTA1-p66Shc interaction impaired actin organization, proliferation, and migration in breast cancer cells. Our results demonstrate a novel reciprocal regulatory mechanism between actin modulation and SNTA1/p66Shc/RhoA signaling cascade in human metastatic breast cancer cells.

## Introduction

Dynamic modulation of the actin cytoskeleton is crucial for most cellular processes. The transition of actin filaments between monomeric (G-actin) and filamentous (F-actin) forms is tightly regulated in cells by several cross-talking signaling networks, involving a diverse range of signaling, scaffolding, and actin-binding proteins (ABPs) (1–4). However, in the case of cancer, the turnover of the actin system is aberrantly regulated. Cancer cells can employ multiple strategies to subvert the delicate and highly coordinated signaling mechanisms that modulate the actin cytoskeleton. The alterations in the actin system in cancer cells mainly arise due to mutation in actin, changes in upstream regulatory proteins, and/or changes in the expression levels of actin-binding proteins (5). In either case, the balance between synergistic and antagonistic activities of actin-binding proteins that regulate actin dynamics is disturbed, consequently enhancing the metastatic phenotype of cancer cells. Although deregulation of the actin system is associated with the pathogenesis of metastatic cancer. However, the mechanism/s that contributes to such transformed invasive phenotype in different cancers is complicated.

Increasing evidence implicates that syntrophin signaling circuitry is crucial for synchronizing the actin cytoskeletal organization in cells (2, 6–8). Alpha-1-Syntrophin (SNTA1) is a 59 kDa multimodular dystrophin-associated peripheral membrane adaptor protein of the syntrophin family (9). It contains two pleckstrin homology (N-terminal PH1 and central PH2) domains, a single PDZ domain, and a highly conserved C-terminal syntrophin unique (SU) domain. These interaction domains of SNTA1 play a crucial role in orchestrating different signaling molecules into various intracellular signaling events (10). SNTA1 has been implicated in regulating several signaling mechanisms linked to the modeling and remodeling of the actin cytoskeleton. Besides directly interacting with F-actin, it also regulates subcellular localization and functioning of various actin organizing proteins like PI3K, PtdIns-4,5-P2, TAPP1, and Rac1, indicating its role in governing actin organization in cells (6, 11–13). Furthermore, cytoskeletal reorganization has been shown to have a role in regulating the subcellular localization of SNTA1 in muscle cells (6). Higher expression of SNTA1has been observed in multiple cancers, including breast, tongue squamous cell carcinomas (SCCs), and colon cancers (14, 15). SNTA1 interacts with Growth factor receptor-bound protein 2 (Grb2) through its tyrosine-phosphorylated proline-rich regions. This interaction facilitates Rac1 GTPase activation that in turn drives cell cycle progression *via* -Sos1-Rac1-PAK1-JNK signaling cascade (12, 16). Recently, we have studied the role of F-actin in the SNTA1 mediated signaling pathway in breast carcinoma cells and loss of SNTA1 tyrosine phosphorylation and decrease in Rac1 activation was observed in response to actin depolymerization (17).

P66shc is a redox adaptor protein that regulates multiple signaling pathways as well as cytoskeletal reorganization. P66Shc is involved in cancer development and it’s overexpressed in various cancers, including metastatic breast cancers (18, 19). P66Shc transduces mitogenic and prosurvival signals through association with Met receptor tyrosine kinase, Grb2, and Grb2-associated binding protein 1 (Gab1). Enhanced expression of p66Shc promotes epithelial-mesenchymal transition (EMT) and migratory phenotype by stimulating Met-dependent signaling and expression of mesenchymal genes in breast cancer cell lines (20). P66Shc has been shown to stimulate cell proliferation in response to growth factor receptor signaling. Upon epidermal growth factor (EGF) stimulation, p66Shc and Grb-2 potentiate cell proliferation, invasion, and migration in breast cancer cells by associating with small GTPases ARF-1 and ARF-6; and coordinating their activation (21). In addition, it has previously been shown that SNTA1/Grb2/p66Shc signaling complex enhances Rac1 activation that in turn augments metastatic characteristics of breast cancer cells (22). Moreover, P66Shc has been observed to localize to focal adhesion complexes where it associates with Focal Adhesion Kinase (FAK). FAK plays a crucial in regulating the activity of small GTPases and downstream effector molecules that mediate cytoskeletal dynamics. The p66Shc/FAK complex facilitates tension-induced RhoA activation in adhesion complexes by activating Rho guanyl exchange factors p115-RhoGEF and GEF-H1 (23, 24).

RhoA GTPase is a well-known regulator of the actin cytoskeleton, which in its active GTP bound state orchestrates actin organization and facilitates diverse cellular responses. RhoA forms part of a signaling cascade where RhoA activates ROCK (Rho-associated coiled-coil containing protein kinase). Active ROCK mediates phosphorylation of LIM kinase, which in turn, negatively regulates cofilin that is involved in F-actin turnover. Furthermore, ROCK also confers activation of myosin light chain kinase (MLC) by inhibiting MLC phosphatase, thus promoting actomyosin assembly (25). Active RhoA also promotes formin activity that mediates activation of profilin which stimulates actin remodeling and regulates various cellular processes like cytokinesis, cell polarity, and migration (25, 26). RhoA has also been shown to bind to lipid proteins like phospholipase D1, PLCG1, and TRPC1 that also bind to SNTA1 (27, 28). RhoA protein has been implicated in cancer progression contributing to cell proliferation, survival, and migration (29, 30). The higher expression or activation of RhoA has been observed in various cancer types such as breast, gastric, head and neck squamous cell carcinoma, and hepatocellular carcinoma (31–33). Taking these findings together, SNTA1/p66Shc mediated signaling pathway seems to have a critical impact on the regulation of Rho GTPase activity and cytoskeletal dynamics in cells, and its dysregulation destabilizes cellular functioning contributing to malignant transformation, tumorigenesis, and metastasis. Here, in this study, we have analyzed the dynamic interplay between actin modulation and SNTA1/p66Shc mediated signaling in regulating cellular events like proliferation, migration as well as cytoskeletal organization in human breast cancer cells. We have used cytochalasin D, a cell-permeable alkaloid, as a tool for altering the dynamics of actin in cells. Cytochalasin D destabilizes the actin cytoskeleton by inhibiting polymerization at the growing end of actin filaments.

## Materials and Methods

### Reagents, Plasmids and Antibodies

Cytochalasin D (C8237), Thiazolyl blue tetrazolium bromide (M2128), Rho activation assay kit (17-294), dimethyl sulfoxide (DMSO) (317275), and phosphatase inhibitors were purchased from Sigma-Aldrich (St. Louis, MO, USA). Protein G plus-agarose beads (IP04), protease inhibitor cocktail (PIC) (539134), and Boyden chamber cell migration kit (ECM 508) were obtained from Millipore (Merck life science, US). The cell culture reagents employed including Dulbecco`s modified Eagle`s medium (DMEM) (11995-065), fetal bovine serum (FBS) (16000-044), penicillin-streptomycin (15140-122), and trypsin (25200-072) were purchased from Gibco, Thermo Fisher Scientific (NY, USA). The polyvinylidene difluoride (PVDF) membrane (GE10600021) was acquired from Amersham biosciences, GE Healthcare (Chicago, IL, US). The Bio-Rad protein estimation kit (500-0006) and protein ladder (PG-PMT2922) were purchased from Bio-Rad Laboratories (USA) and Puregene, Genetix (Asia) respectively. The transfection reagent Lipofectamine 3000 (L3000-008), Alexa Fluor 488 Phalloidin (A12379), antifade mounting solution with NucBlue (Hoechst 33342) (P36983), and Amplex red catalase assay kit (A22180) were purchased from Invitrogen (Carlsbad, CA, US). The wound healing assay kit (CBA-120) and silicone coated coverslips (GBL104112) were obtained from Cell Biolabs, Inc. (San Diego, CA, USA) and Grace-Bio Labs (Bend, OR, US) respectively.

The Xpress-tagged P66Shc construct in pcDNA 3.1 HisA/Xpress and SNTA1-pQB125-myc construct were kindly gifted from Shaida Andrabi (University of Kashmir) and Marvin Adams (University of Washington). The pcDNA 3.1 and SNTA1 vectors were obtained from Addgene (Cambridge, MA, USA). The SYV defective triple mutant construct of p66Shc was previously generated in our lab by the replacement of the C-terminal SYV motif of p66Shc with AFA residues. The SNTA1 double tyrosine mutant construct was mutated at Y^215^ and Y^229^ by phenylalanine residues. Antibody against SNTA1 was purchased from Lifespan Biosciences (1:500, LS-C89921-50) and Invitrogen (1:1000, PA5-22357). Anti-p66Shc (1:1000, PA1-25790, A7F4) and IR- conjugated secondary anti-mouse for the Li-Cor Odyssey-CLx imaging system (DyLight 800, 1:6000, SA535521) were purchased from Invitrogen. Anti-RhoA was obtained from Sigma-Aldrich (1:250, 05-778). Anti-GAPDH and IR-conjugated secondary anti-rabbit (DyLight 800, 1:6000, 5151S) were purchased from Cell Signaling Technology, USA.

### Cell Culture, Transfections and Cytochalasin D Treatment

MDA-MB-231, MCF-7, HBL-100 and T47-D human breast cancer cell lines were obtained from NCCS, India. Cells were cultured with Dulbecco`s modified Eagle`s medium (DMEM) containing 10% heat-inactivated Fetal bovine serum (FBS) and 1% penicillin-streptomycin antibiotic solution at 37°C in a humidified incubator under 5% CO_2_. Stock solution (500 μM) of cytochalasin D was prepared in DMSO. The actin depolymerization was induced by treating cells with different concentrations of cytochalasin D (0.5, 2, 4, and 6 μM) for 6 h. DMSO treated cells were used as vehicle control. For transient transfections, cells were plated for transfection one day prior to the experiment so as to have approximately 60% confluency at transfection. The MDA-MB-231 cells were transfected with desired plasmid constructs using Lipofectamine 3000 as the transfection reagent. Transfection was performed according to the manufacturer`s instructions. The Lipofectamine 3000 reagent was diluted in DMEM in one microcentrifuge tube and the DNA master mix containing plasmid DNA and P3000 reagent in DMEM was prepared in another tube. The DNA mixture was then added in a dropwise manner to the tube containing Lipofectamine 3000 reagent in the ratio of 1:1, followed by incubation at room temperature for 10-15 minutes. The transfected cells were typically analyzed 48 h post-transfection and were harvested or assayed as per protocol.

### Morphological Analysis of Cytochalasin D Treated MDA-MB-231 Cells

MDA-MB-231 cells were plated in 24-well plates at a density of 1.5×10^5^ cells per well and grown overnight to allow attachment. On the next day, they were treated with cytochalasin D (0.5, 2, 4, and 6 μM) for 6 h. DMSO treated cells were used as vehicle control. Morphological changes induced by cytochalasin D in MDA-MB-231 cells were then observed by phase-contrast microscope at ×20 magnification (Floid Imager, Life Technologies).

### Immunofluorescence

To visualize the organization of F-actin, cells grown on coverslips overnight were either transfected with the indicated plasmid constructs and/or treated with cytochalasin D. Cells were washed with 1X PBS followed by fixation with 4% paraformaldehyde in PBS for 15 min at room temperature. Next, cells were washed in PBS, permeabilized with 0.1% Triton X-100 in PBS and blocked with 1% Bovine Serum Albumin (BSA). After permeabilization, cells were washed three times with 1X PBS and incubated in dark for 30 minutes with Alexa Fluor 488-conjugate phalloidin. The coverslips were mounted on glass slides using an antifade mounting solution with NucBlue stain and the samples were visualized using an inverted fluorescence microscope at ×20 magnification (Floid Imager, Life Technologies). The phalloidin-stained F-actin was visualized at the green wavelength (Ex/Em: 482/532 nm) and the nuclei were visualized at the blue wavelength (Ex/Em: 390/446 nm).

### Western Blotting

Cells were lysed in NP-40 lysis buffer (1% NP-40, 150 mM NaCl, 2 mM EDTA, 20 mM Tris-Cl, 10% glycerol) complimented with protease and phosphatase inhibitors (50 mM NaF, 1 mM Na_3_VO_4_, 1 mM PMSF, 10 μl of 1000X PIC stock/ml of lysis buffer). The cell lysates were centrifuged at 10,000 rpm for 20 min at 4°C. The protein concentration of the supernatants was determined by using the Bradford assay. The protein samples were denatured by adding 1X Laemmli sample buffer (50 mM Tris-Cl [pH 6.8], 10% glycerol, 2% SDS, 5% β-mercaptoethanol, 0.01% bromophenol blue) followed by boiling at 100°C for about 5 min. Equal amounts of proteins were loaded onto SDS-PAGE gels and the resolved proteins were electroblotted on PVDF membranes. The blots were then blocked with 5% skim milk or BSA in 1X PBS containing 0.1% Tween-20 (PBS-T) for about 1 hr at room temperature, followed by overnight incubation with specific primary antibodies at 4°C. Afterward, blots were washed three times with chilled 1X TBS containing 0.05-0.1% Tween-20 (TBS-T) and incubated with secondary antibody (DyLight 800) for 1-2 h. The blots were again washed three times with 1X TBS-T, followed by infrared detection using the Li-Cor Odyssey imaging system. Densitometric analysis of the blots was carried out using ImageJ software and data were analyzed using GraphPad Prism software.

### Immunoprecipitation

MDA-MB-231 cells were plated in 10 cm culture dishes at a density of 2×10^6^ cells per plate and cultured overnight in complete medium (DMEM-10% FBS), followed by cytochalasin D treatment. For analyzing protein-protein interaction, immunobeads were prepared by mixing 2-3 μg of the specific primary antibody with 30 μl of protein G-agarose beads. The antibody-bead mixture was kept on a rotator for 4-5 h at 4°C. The immunobeads were washed three times with 1X PBS and equal amounts of protein lysate from the cells treated with or without cytochalasin D were added to it. The mixtures were incubated overnight on a rotator at 4°C, followed by centrifugation at 2000 rpm for 2 min. The pellet containing immunoprecipitated protein complexes was washed three times with 1X PBS, resuspended in 2X sample buffer, and boiled at 100°C for 5 min. The eluted samples were subjected to western blotting as described earlier and analyzed using specific antibodies.

### RhoA Activation Assay

RhoA activation was determined using a Rho activation assay kit that affinity precipitates active RhoA from cell lysates using glutathione conjugated Rhotekin-RBD fusion protein. The assay was performed according to the manufacturer`s instructions. Briefly, MDA-MB-231 cells, either transfected with the indicated plasmid constructs and/or treated with cytochalasin D, were lysed in 1X Mg^2+^ lysis buffer (MLB) with proteases and phosphatase inhibitors. Equal amounts of protein lysates were incubated with 15 μl of GST-RBD agarose beads for 45 min at 4°C on a rocker. Beads were collected by centrifugation (14000×g for 10 sec), washed three times with 1X MLB, and then boiled for 5 min in 2X sample buffer containing 2 μl of 1M dithiothreitol. The active GTP-bound RhoA precipitated with GST-RBD agarose beads was resolved by SDS-PAGE and the RhoA activity was analyzed by western blotting using RhoA specific antibody.

### ROS Generation Assay

The Amplex red catalase assay kit was used for the quantification of extracellular H_2_O_2_ in the cell culture media as per the manufacturer`s protocol. Briefly, cells plated in 6-well plates at a density of ~5×10^5^ cells per well were transfected with appropriate plasmid constructs and/or treated with cytochalasin D. 50 μl of Amplex Red reagent/horseradish peroxide (HRP) working solution (50 μl of 10 mM Amplex Red reagent, 100 μl of 10U/ml HRP stock solution and 4.85 ml of 1X reaction buffer) was added to each well containing transfected, treated and control samples and incubated in dark for 30 min in a CO_2_ incubator. The fluorescence signal was determined by using a spectrofluorophotometer (Shimadzu, RF-5301) with excitation wavelength at 568 nm and emission wavelength at 581 nm.

### Cell Proliferation Assay

The cell proliferation rate was measured using MTT cell proliferation assay. MDA-MB-231 cells cultured in 24-well plates (~1×10^5^ cells per well) were transfected with required plasmid constructs and were finally treated with cytochalasin D, followed by incubation with 200 μl of MTT (5mg/ml) in a humidified atmosphere (37°C, 5% CO_2_) for 3-4 h. Afterward MTT was removed and 200 μl of an acidic DMSO solution was added to each well. Plates were incubated for 30 min in dark on a shaker. The absorbance of the resulting colored solution was measured at 570 nm using a microplate spectrophotometer (Bio-Rad Laboratories, Hercules, CA, USA).

### Wound Healing Assay

Migration of MDA-MB-231 cells transfected with indicated plasmid constructs in the presence or absence of cytochalasin D was analyzed using CytoSelect 24-well wound healing assay kit as per manufacturer`s instructions. Briefly, cells were seeded in a 24-well plate containing plastic inserts at a density of 1×10^5^ cells per well post-transfection with protein of interest. The cells were incubated at 37°C until a monolayer was formed around the insert. The insert was removed from each well leaving a wound gap of 0.9 mm between the cells. The media was gently removed from the wells and cells were washed twice with 1X PBS. This was followed by incubation with cytochalasin D for 6 h. The cells were stained using crystal violet staining and the wound gap was photographed at 0, 12, and 24 h after the start of the assay at ×10 magnification (Olympus CKX41, Life Sciences).

### Transwell Migration Assay

MDA-MB-231 cells were treated with cytochalasin D and/or transfected with wild type and mutant constructs of SNTA1 and p66Shc alone or in combination. The transwell migratory potential of the cells was analyzed utilizing 24-well transwell chemotaxis inserts with semi-permeable porous polycarbonate membrane (8 μm pore size). After appropriate treatment, cells were plated in the upper chamber of the transwell insert at a density of 8×10^4^ cells per well in 300 μl of starvation media. This chamber was placed onto the lower chamber containing media with 10% FBS to generate a chemotactic gradient. After incubating cells for 24 h in an incubator (37°C, 5% CO_2_), media was carefully pipetted out from the insert and the cells present in the upper chamber were gently wiped off using a cotton swab. Finally, the migrated cells present in the lower chamber were stained and destained using the staining and destaining solutions that were provided with the kit and the absorbance of the samples was measured at 565 nm using a standard microplate reader (Bio-Rad Laboratories, Hercules, CA, USA).

### Statistical Analysis

Data were analyzed statistically using Prism 6.07 software (GraphPad software Inc., La Jolla, CA). Each experimental data represents the mean ± standard deviation of three biological replicates. Statistical significance of the mean values was determined using either a one-way or two-way analysis of variance followed by a Dunnett`s or Sidak`s post-test respectively. Asterisks represent statistical significances and are indicated as follows ^*^P<0.05, ^**^P<0.01, ^***^P<0.001.

## Results

### Actin Depolymerization Induced by Cytochalasin D Perturbs SNTA1-p66Shc Interaction in Invasive Breast Cancer Cells

**Figure 1:**
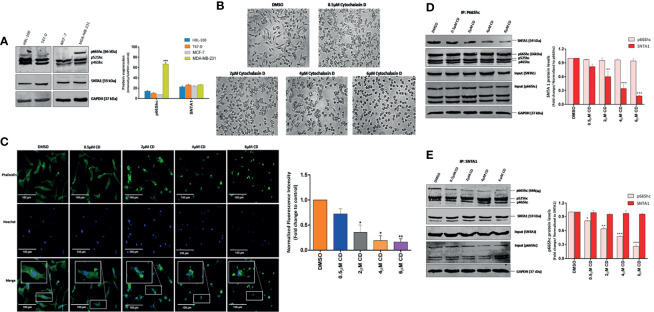
Cytochalasin D mediated actin depolymerization modulates SNTA1/p66Shc interaction in MDA-MB-231 cells. **(A)** Western blot analysis showing endogenous protein expression of p66Shc and SNTA1 in HBL-100, T47-D, MCF-7 and MDA-MB-231 breast cancer cell lines. Anti-GAPDH was used as loading control. The bar graphs show the relative protein expression levels of p66Shc and SNTA1 in HBL-100, T47-D, MCF-7 and MDA-MB-231 breast cancer cells. **(B)** Representative photomicrographs showing the effect Cytochalasin D treatment on morphology of MDA-MB-231 cells. Cytochalasin D (0.5 µM, 2 µM, 4 µM, 6 µM) was added to MDA-MB-231 cells for 6 hours and the morphological characteristics were analyzed by phase contrast microscopy. DMSO was used as vehicle control. Cytochalasin D treated cells adopted sphere-like cell morphology in comparison to normal spindle shaped morphology of control cells. **(C)** Representative fluorescent photomicrographs showing effect of cytochalasin D treatment on F-actin actin organization. Cytochalasin D (0.5 µM, 2 µM, 4 µM, 6 µM) was added to MDA-MB-231 cells for 6 hours and actin cytoskeletal organizations were analyzed by phalloidin-Alexa Fluor 488 staining. Control cells showed uniform F-actin distribution while as cytochalasin D treated cells displayed irregular and punctate F-actin organization. The bar graphs show the fold change in fluorescent intensity of phalloidin stained F-actin in cytochalasin D treated cells in comparison to control. **(D, E)** Endogenous p66Shc and SNTA1 were immunoprecipitated from lysates obtained from cells pretreated with cytochalasin D (0.5 µM, 2 µM, 4 µM, 6 µM) for 6 hours and the protein levels of associated SNTA1 and p66Shc were analyzed by Western blotting respectively. DMSO was used as vehicle control. The densitometric bar graphs of western blots show the fold change in SNTA1 and p66Shc protein levels in p66Shc and SNTA1 immunoprecipitated protein complex respectively. Results shown were repeated three times and are expressed as mean ± SD fold change over control level set to 1 unit and normalized to total protein. The statistical analysis was done using two-way ANOVA followed by Sidak`s multiple comparison test to determine level of significance. *P < 0.05; **P < 0.01; ***P < 0.001.

In order to investigate the role of actin cytoskeletal organization in facilitating SNTA1/p66Shc mediated signaling in breast cancer cells, we first examined the endogenous expression of SNTA1 and p66Shc protein in the MDA-MB-231 (invasive) and HBL-100, T47-D and MCF-7 (non-invasive) breast cancer lines. The expression of SNTA1 protein was found to be similar in these breast cancer cell lines and no significant difference was observed. However, expression of p66Shc protein was restricted only to MDA-MB-231 cells and it was either absent or weakly expressed in MCF-7, HBL-100 and T47-D breast cancer cell lines (**Figure 1A**). This is in consonance with previous studies showing higher expression of p66Shc in this highly invasive metastatic breast cancer cell line (18, 21). Therefore, we selected MDA-MB-231 cell line for our further studies, since it showed higher expression of both the proteins in comparison to other breast cancer cell lines making it a suitable model for studying the involvement of SNTA1/p66Shc mediated signaling in actin cytoskeletal organization and vice versa. Next, we wanted to determine the effect of cytochalasin D mediated actin depolymerization on SNTA1-p66Shc interaction. To investigate this, minimum effective doses of cytochalasin D were determined using MTT assay. MDA-MB-231 cells were treated with increasing concentrations of cytochalasin D (0.5 µM-18 µM) for varying exposure times (3-24 h) at 37°C. Results indicate that cytochalasin D treatment caused a dose-dependent and time-dependent reduction in viability in MDA-MB-231 cells. The dose-responsive curve for cytochalasin D treatment elicits cytotoxicity with an IC_50_ value of 17345 nM and 15822 nM when incubated for 3 and 6 h respectively. Furthermore, the IC_50_ value rapidly lowered to 6854 nM and 5305 nM at 12 and 24 h time points indicating that long term exposure significantly enhances cytochalasin D mediated cytotoxicity in these cells. Therefore, concentrations of cytochalasin D which were much lower than IC_50_ value and an optimum time point of 6 h was selected for this study.

The effect of actin depolymerization on SNTA1-p66Shc interaction was analyzed by treating MDA-MB-231 cells with 0.5 µM, 2 µM, 4 µM and 6 µM concentration cytochalasin D for 6 h. Phase-contrast microscopic observations revealed that cytochalasin D treatment altered cellular morphology inducing cytoplasmic shrinkage and rounding up of the cells. Cytochalasin D treated MDA-MB-231 cells adopted a spherical morphology in contrast to the normal spindle-shaped morphology of control cells (**Figure 1B**). The organization of F-actin in treated and control cells was observed by Fluorescence microscopy. The cells in the control group showed uniform F-actin distribution throughout the cytoplasm and abundant phalloidin staining was observed. In contrast, the F-actin organization was severely disrupted in cytochalasin D treated cells and short punctate fragments of actin were observed. The phalloidin-stained actin cytoskeleton appeared irregular, numerous spinous protrusions emerged at the periphery and a progressive decrease in the fluorescent intensity of F-actin fibers was observed. The fluorescence intensity of phalloidin-stained F-actin substantially declined at or beyond 4 µM cytochalasin D (**Figure 1C**). These changes reveal that cytochalasin D treatment gradually induces F-actin depolymerization and disruption of the actin cytoskeleton. Subsequently, cells were then subjected to co-immunoprecipitation assays and western blot analysis to analyze the effect of cytochalasin D mediated actin filament disruption on SNTA1-p66Shc interaction. As shown in **Figure 1D**, p66Shc co-immunoprecipitated SNTA1 in MDA-MB-231 cells and this association significantly declined in response to cytochalasin D treatment. The levels of SNTA1 protein in the p66shc pull-down complex decreased with increasing concentrations of cytochalasin D and a prominent reduction in the SNTA1 levels was observed at or beyond 4 µM cytochalasin D. These observations were further confirmed by reciprocal co-immunoprecipitation assays. The results here again indicated that cytochalasin D treatment induced a concentration-dependent decrease in p66Shc protein levels in the SNTA1 immunoprecipitated pellet which substantially corroborated our previous findings (**Figure 1E**). These results clearly demonstrate that filamentous actin organization has a role in facilitating physical association between SNTA1 and p66Shc in metastatic breast cancer cells, as the cytochalasin D mediated actin depolymerization destabilized the interaction between these proteins.

### SNTA1, p66Shc and RhoA Form a Trimeric Complex

**Figure 2:**
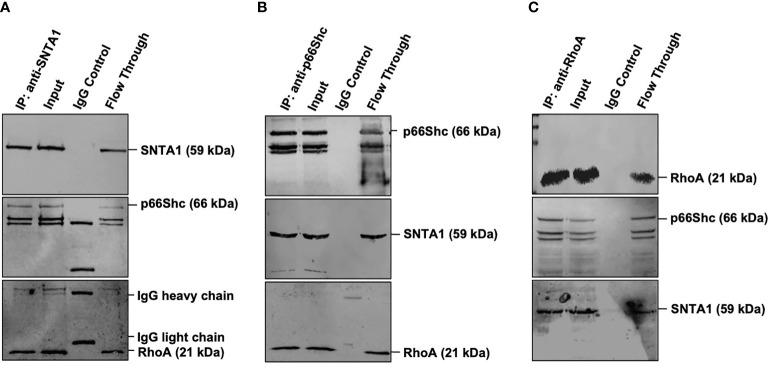
Endogenous SNTA1, p66Shc and RhoA form a stable trimeric complex in MDA-MB-231 cells. **(A)** Co-immunoprecipitation analysis of the interaction between SNTA1, p66Shc and RhoA in MDA-MB-231 cell extracts. IP: immunoprecipitation, Input: total cell lysate, WB: western blot analysis. IgG was used as a control IP. Pull down of the endogenous SNTA1 protein complex was performed using mouse monoclonal SNTA1 antibody. SNTA1, p66Shc and RhoA proteins were detected after western blotting the resulting immunoprecipitates using the rabbit polyclonal SNTA1 antibody, mouse monoclonal Shc antibody and mouse polyclonal RhoA antibody respectively. **(B)** Reciprocal immunoprecipitation analysis of the interaction between p66Shc, SNTA1, and RhoA in MDA-MB-231 cell extracts. IP: immunoprecipitation, Input: total cell lysate, WB: western blot analysis. IgG was used as a control IP. Pull down of the endogenous p66Shc protein complex was done using mouse monoclonal Shc antibody in MDA-MB-231 cells. P66Shc, SNTA1, and RhoA proteins were detected after western blotting the resulting immunoprecipitates using rabbit polyclonal Shc antibody, rabbit polyclonal SNTA1 antibody and rabbit polyclonal RhoA antibody respectively. **(C)** Reciprocal immunoprecipitation analysis of the interaction between RhoA, p66Shc, and SNTA1 in MDA-MB-231 cell extracts. IP, immunoprecipitation; Input, total cell lysate; WB, western blot analysis. IgG was used as a control IP. Pull down of the endogenous RhoA protein complex was done using mouse polyclonal RhoA in MDA-MB-231 cells. RhoA, p66Shc and SNTA1 proteins were detected after western blotting the resulting immunoprecipitates using mouse polyclonal RhoA antibody, rabbit polyclonal Shc antibody and rabbit polyclonal SNTA1 antibody respectively.

RhoA has been shown to interact with the members of the PH domain containing proteins (34–36). In addition to that p66Shc has been previously shown to activate RhoA (23, 24, 37, 38). Since both SNTA1 and p66Shc have been linked with Rho GTPase functioning and cytoskeletal organization, we, therefore, wanted to test whether there is any physical association between SNTA1, p66Shc, and RhoA. The endogenous interaction of SNTA1 with p66Shc and RhoA was determined using co-immunoprecipitation assays. The results from the pulldown assays reveal that SNTA1 interacts with p66Shc and RhoA under physiological conditions in MDA-MB-231 cells. The SNTA1 antibody specifically co-precipitated p66Shc and RhoA proteins in these cells (**Figure 2A**). Moreover, reciprocal co-immunoprecipitation assays were performed to further confirm this association. The reciprocal co-immunoprecipitation analysis indicated that p66Shc antibody co-immunoprecipitated SNTA1 and RhoA protein in MDA-MB-231 cells (**Figure 2B**). Similar observations were made when RhoA was immunoprecipitated from MDA-MB-231 whole cells lysates. The PH domain containing SNTA1 protein and p66Shc were found to be present in RhoA precipitated immunopellets which further confirmed stable multimolecular complex formation between these proteins (**Figure 2C**).

### SNTA1-p66Shc Interaction Mediates ROS Induced RhoA Activation

**Figure 3:**
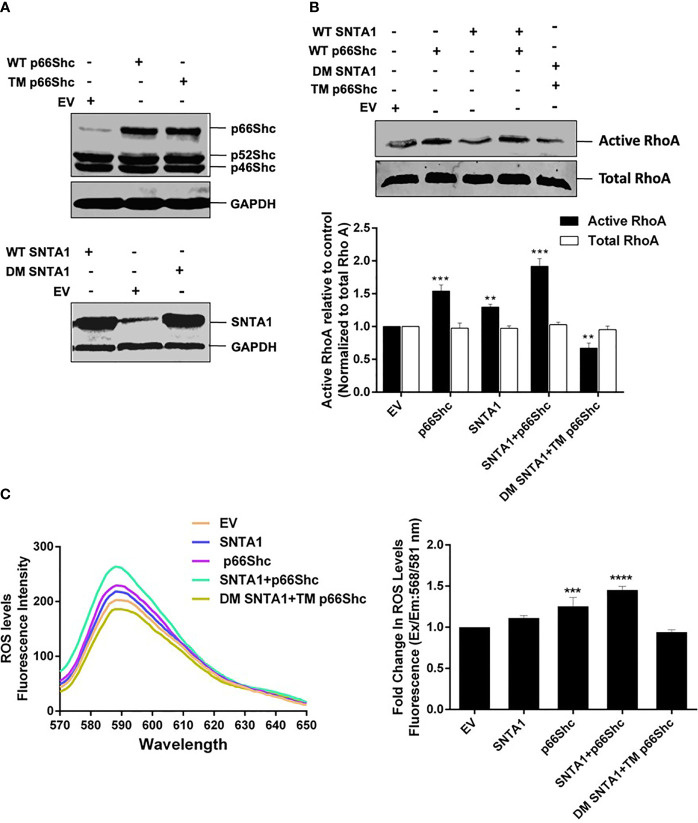
SNTA1-p66Shc interaction is required for ROS induced RhoA activation. **(A)** Representative western blots showing exogenous expression of wild type and mutant forms of p66Shc and SNTA1 protein in MDA-MB-231 cells. GAPDH was used as control. **(B)** Representative western blot showing the relative levels of active RhoA in cells transfected with either vector control (EV) or indicated plasmid constructs. The RhoA activation levels were normalized against total RhoA from whole cell lysates and the relative change in active RhoA levels was determined in comparison to control. The densitometric bar graphs of western blots show the relative change in RhoA protein activation levels in response to over expression of wild type and mutant forms of SNTA1 and p66Shc proteins. Results shown were repeated three times and are expressed as mean ± SD fold change over control level set to 1 unit. The statistical analysis was done using two-way ANOVA followed by Sidak`s multiple comparison test to determine level of significance. **P <0.01;***P < 0.001. **(C)** The emission spectra of amplex red reagent showing the amount of H_2_O_2_ generated in MDA-MB-231 cells over expressing wild type and mutant forms of SNTA1 and p66Shc proteins. The emission spectra were recorded in the wavelength range of 570-650 nm recorded, under an excitation wavelength of 568 nm. The bar graph illustrates the fold change in λ max (fluorescence intensity at 588 nm) in MDA-MB-231 cells over expressing either WT SNTA1, WT p66Shc, WT SNTA1+WT p66Shc (co-expressed), DM SNTA1+TM p66Shc (co-expressed) in comparison to EV control. Results shown were repeated three times and are expressed as mean ± SD fold change over control level set to 1 unit. The statistical analysis was done using one-way ANOVA followed by Dunnett`s multiple comparison test to determine level of significance. ***P < 0.001; ****P <0.0001;

Numerous studies have identified the role of SNTA1 and p66Shc in intracellular ROS generation, and ROS in turn has been implicated in RhoA activation (8, 12, 17, 21, 22, 34–36, 39–43). The functional significance of SNTA1-p66Shc interaction in the regulation of RhoA activation remains unexplored. Therefore, next, in this study, we analyzed the effect of SNTA1-p66Shc interaction on the activity of cellular RhoA using Rhotekin-RBD pulldown assays. MDA-MB-231 cells were transiently transfected with the full-length expression constructs encoding SNTA1 or p66Shc either alone or in combination. Moreover, the consequences of disruption of the SNTA1-p66Shc interaction on RhoA activity in MDA-MB-231 cells were also examined by co-transfecting MDA-MB-231 cells with the mutant plasmid constructs of SNTA1 and p66Shc (DM SNTA1 and TM p66Shc). The C-terminal SYV motif of p66Shc (426-428) and the tyrosine flaking -PXXP- motif of SNTA1 (215-229) are considered to be crucial for the SNTA1-p66Shc interaction, accordingly, SYV and tyrosine defective mutants constructs of p66Shc and SNTA1 respectively were used to perturb interaction between these two proteins. The tyrosine double mutant of SNTA1 (DM SNTA1) represents Y215F and Y229F mutant of SNTA1, where the tyrosine residues flanking the –PXXP- motif have been replaced by phenylalanine. Similarly, the triple mutant of p66Shc (TM p66Shc) represents the SYV dominant-negative mutant of p66Shc, where the C-terminal SYV motif of p66Shc has been replaced by AFA residues. RhoA activation levels in the cells containing ectopically expressed proteins were analyzed in comparison to empty plasmid vehicle (EV) control. Results of the RhoA activation assay revealed that cells transfected with wild-type SNTA1 or wild-type p66Shc showed enhanced RhoA activation compared to control. Furthermore, co-transfection of SNTA1 along with p66Shc synergistically activated RhoA protein and the activation levels were significantly higher in comparison to EV control (**Figures 3A, B**). Conversely, the active RhoA levels were significantly lower in cells co-transfected with tyrosine defective SNTA1 and SYV deficient p66shc mutants (DM SNTA1 and TM p66Shc) in comparison to cells transfected with SNTA1 or p66Shc or when SNTA1 and p66Shc were expressed together. This indicates that the interaction between SNTA1 and p66Shc mediates RhoA activation.

To assess whether the SNTA1-p66Shc mediated enhancement in RhoA activation was a result of ROS generation. We then evaluated the effect of SNTA1-p66Shc interaction on ROS generation in MDA-MB-231 cells using Amplex Red Assays. Our results showed that ROS generation significantly increased in cells that were co-expressing WT SNTA1 and WT p66shc plasmid constructs in comparison to cells that individually expressed WT SNTA1 and WT p66Shc. Furthermore, the ROS levels were significantly declined in cells co-transfected with DM SNTA1 and TM p66Shc in comparison to cells transfected with EV (control), SNTA1, or p66Shc either individually or in combination (**Figure 3C**). These observations are in consensus with the results of the RhoA activation assay as the levels of ROS generation correlated with the RhoA activation levels in MDA-MB-231 cells. Taken together, these findings suggest that SNTA1-p66Shc interaction plays an important role in the regulation of ROS production that consequently facilitates RhoA protein activation.

### Actin Depolymerization Induced by Cytochalasin D Inhibits SNTA1-p66Shc Mediated ROS Generation and RhoA Activation

**Figure 4:**
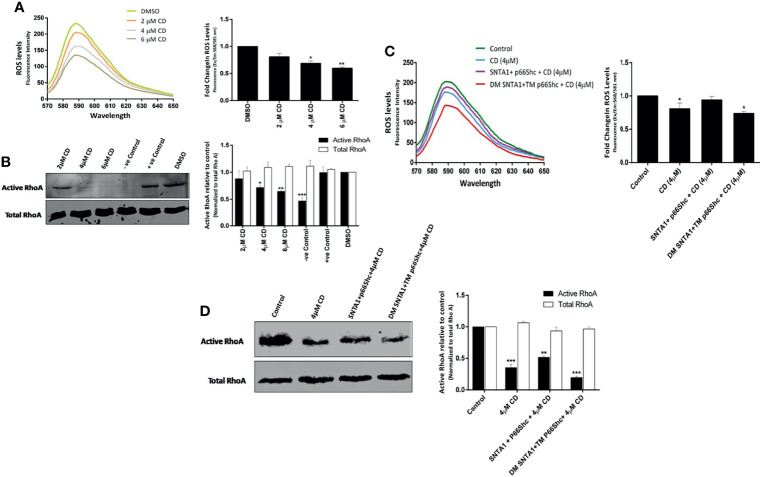
Actin destabilization mediated loss of SNTA1-p66Shc interaction decreases RhoA activation in ROS dependent manner. The ROS and activated RhoA (GTP-bound) levels were determined in MDA-MB-231 cells treated with various concentrations of cytochalasin D (2 µM, 4 µM, 6 µM) and/or with the indicated plasmid constructs. **(A)** The emission spectra of amplex red reagent showing the amount of H_2_O_2_ generated in MDA-MB-231 cells treated with cytochalasin **(D)** ROS levels significantly declined in cytochalasin D treated cells in comparison to control. The emission spectra were recorded in the wavelength range of 570-650 nm recorded, under an excitation wavelength of 568 nm. The bar graph illustrates the fold change in λ max (fluorescence intensity at 588 nm) in MDA-MB-231 cells co-cultured with increased concentrations of cytochalasin **(D)** Results shown were repeated three times and are expressed as mean ± SD fold change over control level set to 1 unit. The statistical analysis was done using one-way ANOVA followed by Dunnett`s multiple comparison test to determine level of significance. *P < 0.05; **P <0.01. **(B)** Representative western blot showing the relative levels of active RhoA in cells treated with cytochalasin **(D)** The RhoA activation levels were normalized against total RhoA from whole cell lysates and the relative change in active RhoA levels was determined in comparison to control. Whole cell lysates pretreated either with GTPγS or GDP prior to precipitation with Rhotekin Rho binding domain were used as positive control and negative control respectively. DMSO was used as vehicle control. RhoA activation assays reveal that cytochalasin D treatment reduces the activation of endogenous RhoA. Densitometry analysis of western blots shows the relative change in RhoA protein activation levels in response to cytochalasin D treatment. Results shown were repeated three times and are expressed as mean ± SD fold change over control level set to 1 unit. The statistical analysis was done using two-way ANOVA followed by Sidak`s multiple comparison test to determine level of significance. *P < 0.05; **P <0.01; ***P < 0.001. **(C)** The emission spectra of amplex red reagent showing the amount of H_2_O_2_ generated in MDA-MB-231 cells transiently transfected with indicated plasmids followed by cytochalasin D treatment. ROS levels were higher in CD- 4 µM/SNTA1-p66Shc (+) cells in comparison to CD- 4 µM and CD- 4 µM/DM SNTA1-TM p66Shc (+) cells. The emission spectra were recorded in the wavelength range of 570-650 nm recorded, under an excitation wavelength of 568 nm. The bar graph illustrates the fold change in λ max (fluorescence intensity at 588 nm) in MDA-MB-231 cells treated with CD- 4 µM post transfection with indicated plasmids. Results shown were repeated three times and are expressed as mean ± SD fold change over control level set to 1 unit. The statistical analysis was done using one-way ANOVA followed by Dunnett`s multiple comparison test to determine level of significance. *P < 0.05. **(D)** Representative western blot showing the relative levels of active RhoA in cytochalasin D treated cells post transfection with wild type and mutant constructs. The RhoA activation levels were normalized against total RhoA from whole cell lysates and the relative change in active RhoA levels was determined in comparison to control. Densitometry analysis of western blots showing relative change in RhoA protein activation levels in transfected and cytochalasin D treated cells. Results shown were repeated three times and are expressed as mean ± SD fold change over control level set to 1 unit. The statistical analysis was done using two-way ANOVA followed by Sidak`s multiple comparison test to determine level of significance. **P <0.01; ***P < 0.001.

Having confirmed that cytochalasin D disrupts SNTA1-p66Shc interaction and the interaction between these two proteins facilitates ROS generation and RhoA activation, we next investigated the effect of actin cytoskeletal modulation on ROS production and RhoA activation to further establish the functional involvement that actin modulation has vis a vis SNTA1-p66Shc interaction and finally ROS induced RhoA activation. MDA-MB-231 cells were treated with various concentrations of cytochalasin D (2 µM, 4 µM, 6 µM) for 6 hours and the levels of ROS and active RhoA were evaluated using Amplex red and RhoA activation assays respectively. The results here indicate that cytochalasin D treatment induced a concentration-dependent decrease in ROS generation and RhoA activation in MDA-MB-231 cells (**Figures 4A, B**). A significant reduction in ROS levels and RhoA activation was observed beyond 2 µM cytochalasin D that is consistent with our previous co-immunoprecipitation results which showed the interaction between SNTA1 and p66Shc decreases in response to cytochalasin D treatment. In addition, cells co-transfected with SNTA1 and p66Shc when treated with cytochalasin D show an increase in ROS generation and RhoA activity in comparison to cells treated with 4 µM cytochalasin D. Conversely, a significant decrease in the levels of ROS and active RhoA was observed in CD-4µM/DM SNTA1-TM p66Shc (+) cells in comparison to CD-4µM/SNTA1-p66Shc (+) and CD-4µM treated cells (**Figures 4C, D**). These results are consistent with the possibility that actin modulation facilitates the SNTA1-P66Shc interaction which in turn mediates ROS production and ultimately change in RhoA activation. Together, these findings suggest that loss of SNTA1-p66Shc interaction mediated by actin depolymerization and/or mutant protein forms downregulates ROS generation and RhoA activity in MDA-MB-231 cells.

### Actin Depolymerization Mediated Loss of SNTA1-p66Shc Interaction and RhoA Activation Impairs Actin Organization, Cell Proliferation, and Migration in Invasive Breast Cancer Cells

**Figure 5:**
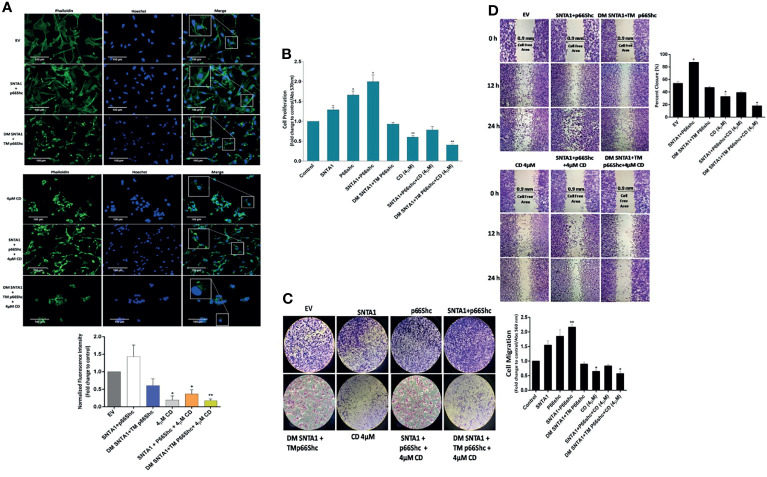
Loss of SNTA1-p66Shc interaction and RhoA activation alters actin organization, cell proliferation and migration in MDA-MB-231 breast cancer cells. **(A)** Representative fluorescent photomicrographs illustrating changes in F-actin actin organization in MDA-MB-231 cells expressing the mentioned plasmid constructs and/or treated with cytochalasin D. The bar graphs show the fold change in fluorescent intensity of phalloidin stained F-actin in comparison to EV control. **(B)** The bar graph shows the proliferative potential of MDA-MB-231 cells expressing the mentioned plasmid constructs and/or treated with cytochalasin D comparison to control. Results shown were repeated three times and are expressed as mean ± SD fold change over control level set to 1 unit. The statistical analysis was done using one-way ANOVA followed by Dunnett`s multiple comparison test to determine level of significance. *P < 0.05; **P <0.01. **(C)** Representative photographs illustrating the difference in migratory potential in cells transfected with mentioned constructs and treated with cytochalasin D in comparison to control. The bar graph depicts fold change in migration in cells transfected with mentioned constructs and treated with cytochalasin D in comparison to control. **(D)** Representative photographs depicting wound healing potential of the cells transfected with mentioned constructs and treated with cytochalasin D in comparison to control. The bar graph shows fold-change in migratory potential in cells transfected with mentioned constructs and treated with cytochalasin D in comparison to control. Results are expressed as mean ± SD from three experiments. The statistical analysis was done using one-way ANOVA followed by Dunnett`s multiple comparison test to determine level of significance. *P < 0.05.

The RhoA signaling plays an active role in modulation of cytoskeletal dynamics, cell adhesion, cell cycle progression, mitosis, and cell migration (44–47). As shown in the results above, SNTA1/p66Shc signaling axis contributed to RhoA activation in metastatic breast cancer cells; whereas cytochalasin D mediated actin depolymerization destabilized this transduction axis reducing RhoA activation levels in these cells. Therefore, we next analyzed the consequences of loss of SNTA1-p66Shc mediated RhoA activation on downstream processes such as actin organization, cell proliferation, and cell migration. The effect of loss of SNTA1-p66Shc interaction and RhoA activation on actin cytoskeletal organization was analyzed *via* fluorescence microscopy using Phalloidin-Alexa Fluor 488 staining on MDA-MB-231 cells. Our results showed that cells co-expressing WT SNTA1 and WT p66Shc displayed regular F-actin cytoskeletal organization. The phalloidin-stained F-actin was evenly distributed throughout the cytoplasm in these cells similar to control group. However, the organization of F-actin was substantially impaired in cells co-transfected with DM SNTA1+TM p66Shc mutant constructs compared to control (**Figure 5A**). The phalloidin-stained actin cytoskeleton in DM SNTA1-TM p66Shc (+) cells appeared irregular and shorter punctate fragments of F-actin were observed. The cytochalasin D treatment was similarly found to induce destabilization and disorganization of normal actin architecture in MDA-MB-231 cells. The fluorescence intensity of phalloidin-stained F-actin markedly declined in these cells in comparison to control. We also observed severe disturbances and disruption of F-actin cytoskeleton in cells that were treated with 4 µM cytochalasin D post transfection with DM SNTA1+TM p66Shc constructs. In contrast, CD-4µM/SNTA1-p66Shc (+) cells resisted cytochalasin D mediated F-actin depolymerization and dysregulation of actin cytoskeletal architecture and fluorescence intensity of phalloidin stained F-actin was significantly higher in these cells compared to CD-4µM treated cells. These results suggest that SNTA1-p66Shc interaction is important in maintenance of normal actin cytoskeletal arrangement in MDA-MB-231 cells. The proliferation levels were determined in cells overexpressing SNTA1, p66Shc, SNTA1+p66Shc, DM SNTA1+TM p66Shc alone or in combination with cytochalasin D using MTT assay. Results showed that overexpression of SNTA1 and p66Shc increased proliferation in MDA-MB-231 cells compared to control cells and a maximal increase in cell proliferation was observed when both SNTA1 and p66Shc were expressed together (**Figure 5B**). A significant decrease in proliferation was observed in DM SNTA1+TM p66Shc (+) cells where the SNTA1-p66Shc interaction was disrupted by the use of mutant constructs. Comparison of the CD-4µM treated cells with CD-4µM/SNTA1+p66Shc (+) and CD-4µM/DM SNTA1+TM p66Shc (+) cells showed the cells co-expressing SNTA1+p66Shc when treated with cytochalasin D displayed an increase in proliferation whereas a decrease in proliferation levels was observed in cells treated with cytochalasin D post-transfection with DM SNTA1+TM p66Shc mutant constructs as compared to cells treated with cytochalasin D alone. These results demonstrate that loss of SNTA-p66Shc interaction impairs remodeling of actin cytoskeleton and a possible disturbance of actin cytoskeleton consequently results in reduced proliferation levels of MDA-MB-231 cells.

We then analyzed the migratory potential of MDA-MB-231 cells treated with cytochalasin D and/or transfected with wild-type and mutant constructs of SNTA1 and p66Shc alone or in combination using transwell assays and wound healing assays. In Boyden chamber transwell assay, SNTA1 and p66Shc co-expression significantly increased the migration of MDA-MB-231 cells compared with the cells expressing SNTA1 and p66Shc alone. Moreover, cells co-expressing DM SNTA and TM p66Shc mutant construct depicted decreased migratory potential in comparison to EV control, SNTA1, p66Shc, SNTA1+p66Shc expressing cells. Also, cytochalasin D treatment considerably lowered the migratory potential of MDA-MB-231 cells in comparison to control. MDA-MB-231 cells co-expressing SNTA1 and p66Shc when treated with 4µM cytochalasin D showed enhanced cell migration in comparison to cells treated with 4µM cytochalasin D alone, while as the cells co-expressing DM SNTA1 and TM p66Shc when treated with 4µM CD depicted decreased migration in comparison to CD-4µM treated cells (**Figure 5C**). Similarly, we examined the migratory behavior of the cells co-expressing wild type and mutant constructs of SNTA1 and p66Shc alone or in combination with cytochalasin D by wound healing assay. Results of the wound healing assay revealed that cell migration into the wound gap was marginally declined in CD-4µM/DM SNTA1+TM p66Shc (+) cells and CD-4µM cells when compared to their control counterparts. Co-expression of SNTA1 and p66Shc significantly enhanced the percent of wound closure in MDA-MB-231 cells compared with the EV control and the closure of wound gap in SNTA1-p66Shc (+) cells was observed after 12 and 24 hours of wounding (**Figure 5D**). The transwell chamber and wound healing assays both suggest that loss of SNTA1-p66Shc interaction decreases the migration potential of MDA-MB-231 breast cancer cells. These findings point towards the possible role of the actin cytoskeleton in facilitating SNTA1-p66Shc mediated migration *via* RhoA protein activation in these cells.

## Discussion

Alpha-1-syntrophin is one of the well-established regulators of cell signaling and actin organization (2, 6, 7, 13, 39, 48). SNTA1 functions as a scaffolding adaptor protein that integrates actin cytoskeleton and internal signaling apparatus to the extracellular matrix *via* Dystrophin Glycoprotein complex (6, 8, 12). Elevated expression of SNTA1 and its enhanced signaling has been shown to be associated with breast cancer progression; however, the underlying mechanism remains unclear. Here, we demonstrate a crucial role for the actin cytoskeletal modulation in driving SNTA1/p66Shc signal transduction mediated pro-metastatic phenotypes in breast cancer cells. Using immunoprecipitation assays, we found that SNTA1 and p66Shc form a stable complex in metastatic breast cancer cells, and cytochalasin D mediated actin filament depolymerization decreased the endogenous physical association between these proteins. This observation is consistent with the previous reports which suggested SNTA1/GRB2/p66Shc signaling axis propagates tumorigenic signals in breast cancers (22, 42, 49, 50). Emerging evidence indicate that SNTA1 and p66Shc can simultaneously couple to GRB2 *via* specific phosphotyrosine motifs. The tyrosine flanking proline-rich -PXXP- motif (Y^215^ and Y^229^) of SNTA1 as well as SYV (426-428) motif of p66Shc have been shown to bind to GRB2 upon phosphorylation (12, 51). Besides, previously we have also found that actin filament destabilization attenuates SNTA1 tyrosine phosphorylation and impedes its functioning in breast cancer cells (17, 52). The present data clearly indicates that actin cytoskeleton is involved in maintenance of SNTA1-p66Shc interaction in metastatic breast cancer cells. Indeed, we observed a concentration-dependent decrease in SNTA1-p66Shc interaction in response cytochalasin D mediated actin filament destabilization.

Although, SNTA1 signaling pathway has been implicated in dynamic remodeling of actin cytoskeleton, but the precise mechanism/s involved in SNTA1 mediated actin modulation in metastatic breast cancers are still unknown. Here, we found that SNTA1/p66Shc/RhoA form a stable trimeric complex at an endogenous level in MDA-MB-231 cells. Importantly, we observed that SNTA1/p66Shc signaling axis positively regulates RhoA activity in this multiprotein signaling complex. RhoA GTPase is an important modulator of actin dynamics. (45, 53, 54). RhoA dysregulation is observed in breast cancers contributing to progression and metastasis (47). Recent studies have also shown the dynamic involvement of p66Shc in actin reorganization and RhoA activation (23, 37, 38). Furthermore, PH domain containing proteins have been reported to be involved in modulation of RhoA functioning (34–36). Our data is consistent with the possibility that SNTA1/p66Shc signaling module couples to RhoA to modulate its activity in MDA-MB-231 cells. The dominant negative SYV mutant form of p66Shc and dominant negative Y^215^/Y^229^ mutant form of SNTA1 significantly inhibited RhoA activation. In contrast, co-expression of SNTA1 and p66Shc enhanced RhoA activation levels in comparison to cells over-expressing SNTA1 or p66Shc alone. Moreover, we observed SNTA1 and p66Shc positively regulates ROS generation. A significant increase in ROS generation was observed on co-transfecting cells with SNTA1 and p66Shc plasmid constructs. However, the ROS levels marginally declined in cells co-expressing DM SNTA1 and TM p66Shc mutant proteins. These results indicate that SNTA1 and p66Shc act synergistically to contribute to ROS generation. Several reports have demonstrated the direct involvement of ROS in RhoA activation (55–58). RhoA protein has been shown to contain a redox sensitive motif (GXXXCGK(S/T)C) which in response to ROS stimulates guanine nucleotide exchange activity and upregulates RhoA activation (59, 60). We propose that SNTA1/p66Shc signaling stimulates ROS production that consequently facilitates RhoA activation in MDA-MB-231 cells. In addition, we analyzed the role of actin cytoskeletal reorganization on SNTA1-p66Shc signaling mediated ROS generation and RhoA activation by treating cells with cytochalasin D. Our results demonstrate that the levels of active RhoA as well ROS generation declined in response to actin depolymerization. This is in corroboration with the earlier observation that showed loss of SNTA1-p66Shc interaction is triggered by cytochalasin D treatment. We assume that the decrease in ROS and active RhoA levels is likely due to disruption of SNTA1-p66Shc interaction. Our assumption was further substantiated by the finding that the cytochalasin D mediated decrease in ROS production and RhoA activation was rescued in cells co-transfected with SNTA1 and p66Shc plasmids. These results demonstrate the novel role of actin cytoskeletal modulation facilitate SNTA1-p66Shc interaction that consequently triggers ROS generation and RhoA activation in breast cancer cells.

**Figure 6:**
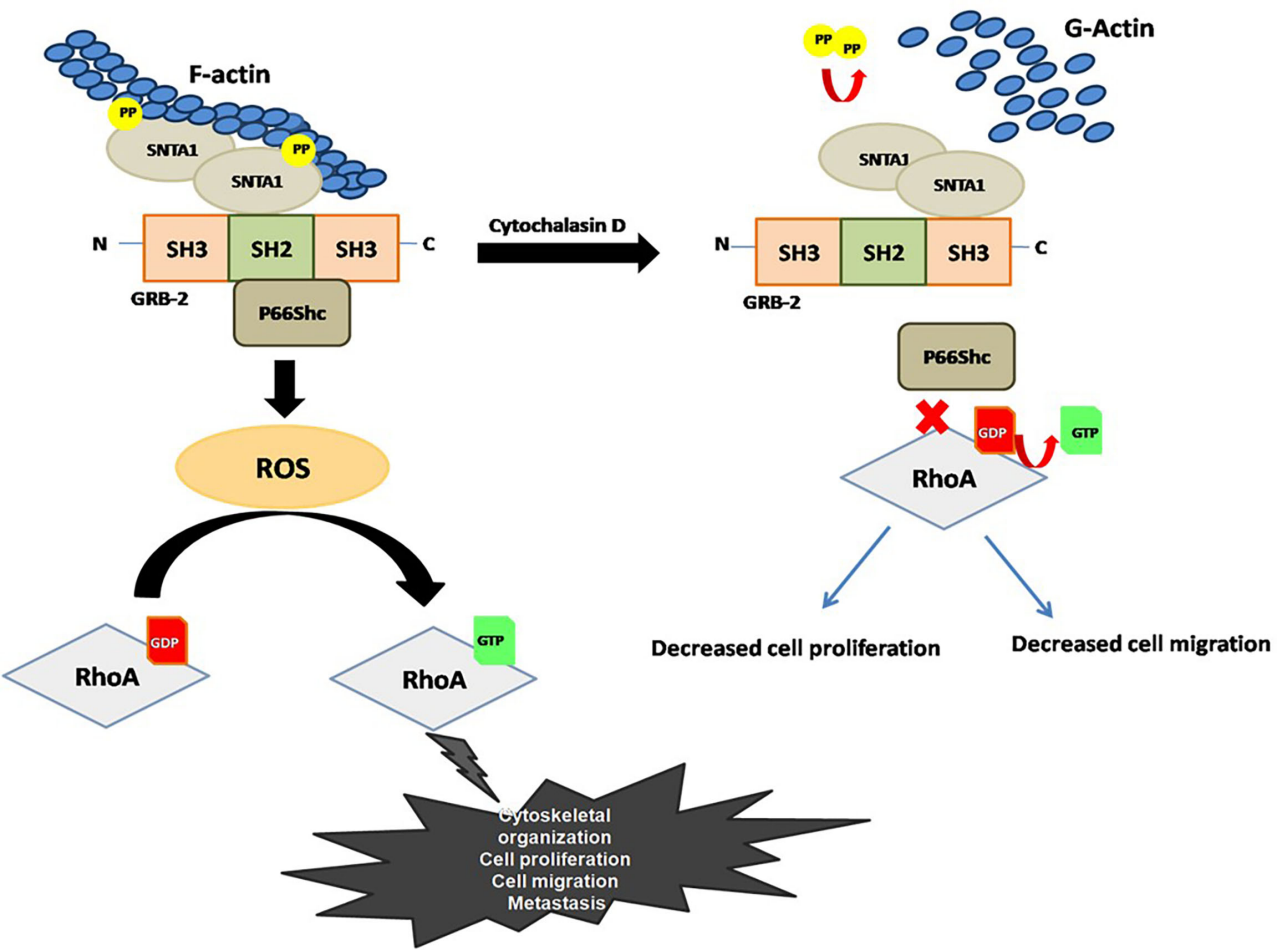
Proposed model for the role F-actin in the regulation of SNTA1-p66Shc mediated RhoA activity in metastatic breast cancer cells.

We observed that SNTA1/P66Shc/RhoA signaling downregulation altered actin organization, cell proliferation as well as decreased the migratory potential in breast cancer cells. Our result shows that co-expression of SNTA1 and p66Shc significantly enhanced the migratory and proliferative potential of cells in comparison to cells expressing SNTA1 and p66Shc alone. Moreover, cells over-expressing DM SNTA1 and TM p66Shc mutants together or treated with cytochalasin D showed irregular actin organization and decline in cell migration and proliferation was also observed. Interestingly, when MDA-MB-231 cells were treated with cytochalasin D post transfection with SNTA1 and p66Shc simultaneously, the inhibitory effect of cytochalasin D on these cellular processes overcame to some extent, pointing to the reciprocal role of actin cytoskeletal dynamics and SNTA1-P66Shc interaction in these cellular processes. Finally, we did a preliminary *in silico* analysis of SNTA1, p66Shc and RhoA proteins in The Cancer Genome Atlas (TCGA) in the breast cancer cohort, in order to confirm the prognostic value of the proteins in this study. We found that there were 24 mutations in total as well as differential expression in our proteins of interest in the breast site mutational analysis data of TCGA. The database for this *in silico* analysis revealed that SNTA1 and RhoA showed multiple deleterious mutations in breast cancers, which ultimately were shown to have a damaging impact. The mutations found in p66Shc were shown to be mainly tolerated and hence benign. Moreover, the expression levels of all the three proteins were found to be altered in case of cancer samples as compared to the controls. This information from the TCGA confirms the prognostic potential of SNTA1, p66Shc as well as RhoA protein (**Supplementary Tables 1**, **2**). In summary, we have demonstrated reciprocal regulation between actin modulation and SNTA1/p66Shc/RhoA signaling cascade in human metastatic breast cancer cells. As shown in the **Figure 6**, Actin modulation affects the SNTA1/p66Shc signaling cascade, contributing to ROS induced RhoA activation and RhoA driven downstream cellular events. Conversely, actin cytoskeletal destabilization perturbs SNTA1-p66Shc signaling mediated ROS generation and RhoA activation thereby inhibiting cell proliferation and migration. Knowledge driven from this mechanistic signaling can be exploited for prognostics and therapeutics on breast cancers.

## Data Availability Statement

The raw data supporting the conclusions of this article will be made available by the authors, without undue reservation.

## Author Contributions

The work was done under the supervision of SS and FK. SS and FK planned all the experiments and finalized the manuscript. RA did most of the experiments and drafted the Manuscript. HM helped with some of the immunoprecipitation and western blotting experiments. RH helped in drafting the manuscript and helped in the ROS assay experiments. BB carried out the *in-silico* analysis. RS helped with the cell culture. All authors contributed to the article and approved the submitted version.

## Funding

This work was supported by grant to Sahar Saleem Bhat (No.DST/INSPIRE/04/2016/001373) by the Department of Science and Technology (DST), India and to RA (No.09/251(0086)/2017-EMR-I) by Council of Scientific and Industrial Research (CSIR), India.

## Conflict of Interest

The authors declare that the research was conducted in the absence of any commercial or financial relationships that could be construed as a potential conflict of interest.

## Publisher’s Note

All claims expressed in this article are solely those of the authors and do not necessarily represent those of their affiliated organizations, or those of the publisher, the editors and the reviewers. Any product that may be evaluated in this article, or claim that may be made by its manufacturer, is not guaranteed or endorsed by the publisher.
