# Supplementary material for: Actin Modulation Regulates the Alpha-1-Syntrophin/p66Shc Mediated Redox Signaling Contributing to the RhoA GTPase Protein Activation in Breast Cancer Cells
Source: Front Oncol. 2022 Feb 21;12:841303. doi: 10.3389/fonc.2022.841303 (PMC8904154; doi:10.3389/fonc.2022.841303)
Supplement: Supplementary file 1 [file Table_1.docx]

Supplementary table 1. Expression analysis of RhoA protein, SNTA1 protein and p66Shc (SHC1) protein as done by TCGA breast cancer data

| \| **Gene Name** \| \| --- \| | **No. Of samples** | **Experiment accession** | **Comparison** | **Log2(FC)** | **Adjusted p-value** |
| --- | --- | --- | --- | --- | --- | --- |
| RHOA | 6 | E-MTAB-5537 | estrogen receptor alpha shRNA' vs 'scrambled shRNA' | 1.8 | 1.82E-31 |
| RHOA | 56 | E-GEOD-10797 | stromal cell; invasive breast cancer' vs 'epithelial cell; invasive breast cancer' | -1.8 | 0.000374595 |
| RHOA | 8 | E-GEOD-49608 | '7 hour' vs '0 hour' in 'MDA-LM2' | 1.2 | 0 |
| RHOA | 9 | E-GEOD-49608 | '7 hour' vs '0 hour' in 'MDA-MB-231' | 1 | 1.83E-136 |
| SNTA1 | 6 | E-MTAB-5537 | 'estrogen receptor alpha shRNA' vs 'scrambled shRNA' | 3.3 | 1.39E-47 |
| SNTA1 | 6 | E-GEOD-68086 | breast carcinoma' vs 'normal' | -2.2 | 3.29E-07 |
| SNTA1 | 27 | E-MTAB-779 | 'fibroadenomatosis' vs 'normal' | -1.6 | 0.024633744 |
| SNTA1 | 42 | E-MTAB-779 | 'breast carcinoma' vs 'normal' | -1.5 | 0.000139508 |
| SNTA1 | 28 | E-MTAB-779 | 'fibroadenoma' vs 'normal' | -1.4 | 0.034084004 |
| SNTA1 | 7 | E-MTAB-3021 | 'PRPF8' vs 'control' | -1.2 | 2.15768E-06 |
| SNTA1 | 6 | E-MTAB-6647 | 'hypoxia' vs 'normoxia' in 'BT549; Plasmax media' | 1.1 | 1.21E-12 |
| SNTA1 | 6 | E-GEOD-58326 | 'siZNF217' vs 'control' | -1 | 3.09914E-06 |
| SNTA1 | 6 | E-GEOD-63452 | '24 hour' vs '0 hour' in 'MCF-10A' | 1 | 0.02498521 |
| SHC1 | 17 | E-GEOD-52194 | 'HER2 Positive Breast Carcinoma; breast carcinoma' vs 'normal' | -1.8 | 1.27177E-05 |
| SHC1 | 7 | E-MTAB-3021 | 'PRPF8' vs 'control' | 1.4 | 1.07E-39 |
| SHC1 | 6 | E-MTAB-688 | 'MOF siRNA' vs 'control siRNA' in 'FOXP3 expression' | 1.4 | 1.97798E-06 |
| SHC1 | 9 | E-GEOD-52194 | 'non-triple-negative breast cancer; breast carcinoma' vs 'normal' | -1.4 | 0.000269344 |
| SHC1 | 6 | E-MTAB-5537 | 'estrogen receptor alpha shRNA' vs 'scrambled shRNA' | 1.3 | 1.67E-17 |
| SHC1 | 6 | E-MTAB-688 | 'MOF siRNA' vs 'control siRNA' in 'doxycycline-mediated repression of FOXP3' | 1.3 | 1.17268E-05 |
| SHC1 | 6 | E-GEOD-42781 | 'delta-her2 overexpression' vs 'control' | 1.2 | 3.45867E-05 |
| SHC1 | 8 | E-GEOD-41678 | 'Cal51; cocultured with fibroblast HFF1' vs 'Cal51; normal' | 1.1 | 1.81E-07 |
| SHC1 | 6 | E-GEOD-27473 | 'estrogen receptor alpha knockdown' vs 'control' | 1.1 | 1.27169E-05 |
| SHC1 | 26 | E-GEOD-7515 | 'mammosphere cell' vs 'breast tumor cell' | 1.1 | 0.001182303 |
| SHC1 | 56 | E-GEOD-10797 | 'stromal cell; invasive breast cancer' vs 'epithelial cell; invasive breast cancer' | -1.1 | 0.034630285 |
| SHC1 | 6 | E-GEOD-52707 | 'SKR6LR cells resistant to lapanitib' vs 'SKR6 cells with elevated HER2 expression' | 1 | 0.000504019 |
| SHC1 | 8 | E-GEOD-52194 | 'triple-negative breast cancer; breast carcinoma' vs 'normal' | -1 | 0.033110787 |
| SHC1 | 6 | E-GEOD-69540 | 'neuregulin' vs 'none' at '24 hour' | 1 | 0.048521417 |

Supplementary table 2: Mutation analysis of RhoA gene, SNTA1 gene and SHC1 (p66Shc) gene as done by TCGA data; site breast

| **Base pair change** | **Type** | **Mutation Type - AA Change** | **No. of cases** | **Effect** | **Sift Score / Impact** | **PolyPhen Score / Impact** |
| --- | --- | --- | --- | --- | --- | --- |
| **Gene Name: RhoA** | | | | | | |
| chr3:g.49375472C>G | Substitution | Missense - E40Q | 3 | MODERATE | 0.04 / deleterious | 0.534 / possibly_damaging |
| chr3:g.49362531C>G | Substitution | Missense - E125Q | 1 | MODERATE | 0.32 / tolerated | 0.011 / benign |
| chr3:g.49375540C>G | Substitution | Missense - G17A | 1 | MODERATE |  | 0.591 / probably_damaging |
| chr3:g.49360355C>G | Substitution | Missense - D146H | 1 | MODERATE | 0.11 / tolerated | 0.01 / benign |
| chr3:g.49362550C>G | Substitution | Missense - K118N | 1 | MODERATE |  | 0.985 / probably_damaging |
| chr3:g.49360367C>T | Substitution | Missense - E142K | 1 | MODERATE | 0.05 / tolerated | 0.089 / benign |
| chr3:g.49360229_49360230insT | Insertion | Frameshift - S188Ifs*30 | 1 | HIGH |  |  |
| chr3:g.49360382C>T | Substitution | Missense - E137K | 1 | MODERATE | 0.47 / tolerated | 0.006 / benign |
| chr3:g.49360170C>G | Substitution | 3 Prime UTR | 1 | MODIFIER |  |  |
| chr3:g.49375524G>C | Substitution | Synonymous - L22L | 1 | LOW |  |  |
| chr3:g.49375577G>A | Substitution | Missense - R5W | 1 | MODERATE | 0.02 / deleterious | 0.007 / benign |
| **Gene Name: SNTA1** | | | | | | |
| chr20:g.33408512C>A | Substitution | Missense - A505S | 1 | MODERATE | 0.01 / deleterious | 0.998 / probably_damaging |
| chr20:g.33408403T>C | Substitution | 3 Prime UTR | 1 | MODIFIER |  |  |
| chr20:g.33408475delC | Deletion | 3 Prime UTR | 1 | MODIFIER |  |  |
| chr20:g.33412702delGCCCACGACCTCGCACTA | Deletion | Inframe Deletion - S256_A261del | 1 | MODERATE |  |  |
| **Gene Name: SHC1 (p66Shc)** | | | | | | |
| chr1:g.154970222G>C | Substitution | Stop Gained - S102* | 1 | HIGH |  |  |
| chr1:g.154966204G>C | Substitution | Missense - P404A | 1 | MODERATE | 0.54 / tolerated | 0.004 / benign |
| chr1:g.154965639C>T | Substitution | Synonymous - L510L | 1 | LOW |  |  |
| chr1:g.154964237G>A | Substitution | Intron - | 1 | MODIFIER |  |  |
| chr1:g.154970206G>A | Substitution | Synonymous - L107L | 1 | LOW |  |  |
| chr1:g.154966053G>C | Substitution | Missense - S427C | 1 | MODERATE | 0.31 / tolerated | 0.037 / benign |
| chr1:g.154963887G>A | Substitution | Synonymous - I557I | 1 | LOW |  |  |
| chr1:g.154970341G>A | Substitution | Synonymous - F62F | 1 | LOW |  |  |
| chr1:g.154963788G>C | Substitution | 3 Prime UTR - | 1 | MODIFIER |  |  |
| chr1:g.154969388G>C | Substitution | Missense - Q186E | 1 | MODERATE |  | 0.376 / benign |
